# Indeterminate Domain Proteins Regulate Rice Defense to Sheath Blight Disease

**DOI:** 10.1186/s12284-020-0371-1

**Published:** 2020-03-06

**Authors:** Qian Sun, Dan Dan Li, Jin Chu, De Peng Yuan, Shuang Li, Li Juan Zhong, Xiao Han, Yuan Hu Xuan

**Affiliations:** 1 College of Plant Protection, Shenyang Agricultural University, Shenyang, 110866 China; 2 Institute of Plant Protection, Liaoning Academy of Agricultural Sciences, Shenyang, 110161 China; 3 Shaanxi Key Laboratory of Chinese Jujube, Yan’an University, Yan’an, 716000 Shaanxi China; 4 College of Life Science, Yan’an University, Yan’an, 716000 Shaanxi China; 5 Microbial Research Institute, Liaoning Academy of Agricultural Sciences, Chaoyang, 122000 China; 6 College of Biological Science and Engineering, Fuzhou University, Fuzhou, 350108 China

**Keywords:** Indeterminate domain protein, Sheath blight disease, Transcription activation, Defense, Rice

## Abstract

**Background:**

Loose Plant Architecture 1 (LPA1), an indeterminate domain (IDD) protein, exhibits almost no expression in the leaves, but the overexpression of *LPA1* significantly increases the resistance of rice to sheath blight disease (ShB) via the activation of *PIN-FORMED 1a* (*PIN1a*).

**Results:**

In this study, we determined that *Rhizoctonia solani* infection significantly induced *LPA1* expression in the leaves, and *lpa1* was more susceptible to *R. solani* compared with the wild-type and revertant plants. In addition, infection with *R. solani* altered the expression of *IDD3*, *IDD5*, *IDD10*, and *IDD13*, and yeast two-hybrid, split-GFP, and coimmunoprecipitation assays showed that LPA1 interacts with IDD3 and IDD13. *IDD13 RNAi* plants were more susceptible, while *IDD13* overexpressors were less susceptible to ShB compared with the wild-type. In parallel, *idd3* exhibited no significant differences, while *IDD3* overexpressors were more susceptible compared to the wild-type response to ShB. Additional chromatin-immunoprecipitation and electrophoretic mobility shift assay experiments indicated that IDD13 and IDD3 bound to the *PIN1a* promoter, and the transient assay indicated that IDD13 and IDD3 positively and negatively regulate *PIN1a* expression, respectively. Moreover, IDD13, IDD3, and LPA1 form a transcription factor complex that regulates *PIN1a*. A genetic study showed that the *LPA1* repressor lines were similar to *lpa1/IDD13 RNAi* and were more susceptible than the *lpa1* and *IDD13 RNAi* plants in response to ShB. The overexpression of *IDD13* increased resistance to ShB in the *lpa1* background.

**Conclusions:**

Taken together, our analyses established that IDD3, IDD13, and LPA1 form a transcription factor complex to regulate the defense of rice against ShB possibly via the regulation of *PIN1a*.

## Background

Sheath blight disease (ShB) is one of the three major diseases that are caused by *Rhizoctonia solani* in rice (*Oryza sativa*) (Savary et al., 1995). The fungus damages rice during the whole period of the growth cycle and primarily infects the leaves, sheaths, and panicles. At the late stage of infection, the whole plant withers and lodges (Savary et al., 1995). ShB can reduce rice yield production up to 50% when the disease is severe (Savary et al. 2000). Since there is a lack of resistant cultivars against ShB, the application of fungicides is the current primary approach to control this disease (Savary et al. 2000). However, its use severely influences environmental conditions because of its effect on microbes in the environment, and the fungicides also increase the cost of cultivation. Thus, the isolation of resistant rice cultivars and the exploration of defense mechanisms against SbB have become an important issue. Previous studies have demonstrated that the overexpression of chitinase, β-1,3-glucanase, or polygalacturonase-inhibiting protein (OsPGIP1) enhances the resistance of rice to *R. solani* (Shah et al. 2009; Mao et al. 2014; Wang et al. 2015). The overexpression of an ethylene synthesis enzyme (OsACS2) promotes the resistance of rice to blast and sheath blight (Helliwell et al. 2013). The overexpression of BROAD-SPECTRUM RESISTANCE2 (BSR2) resulted in resistance to *R. solani* in Arabidopsis and rice (Maeda et al. 2019), and salicylic acid-dependent immunity contributes to resistance against *R. solani* in rice and *Brachypodium distachyon* (Kouzai et al. 2018). In addition, our recent studies identified that a mutation in *Sugar Will be Eventually be Exported Transporter 11* (*SWEET11*) significantly promoted the defense of rice to ShB (Gao et al. 2018); Related to ABI3/VP1-Like 1 (RAVL1) modulates rice defense against ShB via the activation of brassinosteroids and ethylene signaling genes (Yuan et al. 2018), and the overexpression of *LPA1* (*IDD14*) promoted the defense of rice against ShB via the activation of *PIN1a* (Sun et al. 2019).

The indeterminate domain (IDD) consists of two C_2_H_2_ and two C_2_HC zinc finger motifs, and the IDD genes play diverse biological functions in plants. ID1 has been reported to control the flowering time in maize and rice (Colasanti et al. 1998; Park et al. 2008). Magpie (MAG)/AtIDD3 and jackdaw (JKD)/AtIDD10 regulate the fate of root cells (Welch et al. 2007). Enhydrous (ENY)/AtIDD1 regulates seed maturation (Feurtado et al. 2011). AtIDD8 modulates plant development (Seo et al. 2011). AtIDD14, AtIDD15, and AtIDD16 cooperatively regulate lateral organ morphogenesis and gravitropism by promoting auxin biosynthesis and transport in Arabidopsis (Cui et al. 2013). Loose plant architecture1 (LPA1)/IDD14 regulates shoot gravitropism and lamina joint angle (Wu et al. 2013; Liu et al. 2016). The regulator of CBF1 (ROC1)/IDD3 activates DREB1B/CBF1 to regulate chilling tolerance in rice (Dou et al. 2016). IDD2 regulates secondary cell wall formation in rice (Huang et al. 2018). In addition, the AtIDD4 repressor constitutively induces immunity in Arabidopsis (Volz et al. 2019). The binding motifs of the transcription factor IDD have been identified in maize (ID1, 5′-TTTGTC^G^/_C_TTTT-3′), Arabidopsis (AtIDD8, 5′-TTTTGTCC-3′), and rice (IDD10, 5′-TTTGTC^C^/_G_) (Kozaki et al. 2004; Seo et al. 2011; Xuan et al. 2013). However, the function of IDD in plant defense, as well as the IDD target genes, remains largely unknown.

Auxin is one of the key phytohormones, and its polar transport is regulated by auxin influx AUX1/LAX and efflux protein PINs (Adamowski and Friml, 2015; Zazimalova et al. 2010). Auxin plays key roles in plant growth and development, as well as in controlling plant defense (Robert-Seilaniantz et al. 2011; Naseem et al. 2012; Chen et al. 2007). More studies identified that auxin signaling regulates rice defense against the bacterial pathogen *Xanthomonas oryzae* (Fu et al. 2011) and the fungal pathogen *Magnaporthe oryzae* (Fu et al. 2011). Recently, we identified that exogenously treated auxin increases the resistance of rice to *R. solani* AG1-IA and revealed that *LPA1* overexpression activates *PIN1a* to promote defense against *R. solani* in rice (Sun et al. 2019). However, whether other IDDs regulate the resistance of rice to ShB remains to be elucidated. In this study, we performed molecular, biochemical, and genetic studies to explore the function of IDD in rice defense. The results showed that IDD3 and IDD13 interact with LPA1 to regulate *PIN1a* expression and act to modulate the resistance of rice to ShB. Taken together, our analyses provide information on the role of the IDDs in the regulation of rice defense, as well as the regulatory mechanism for ShB in rice.

## Results

### *LPA1* Is Induced by *Rhizoctonia solani*, and *lpa1* Is more Susceptible to Sheath Blight Disease

**Fig. 1:**
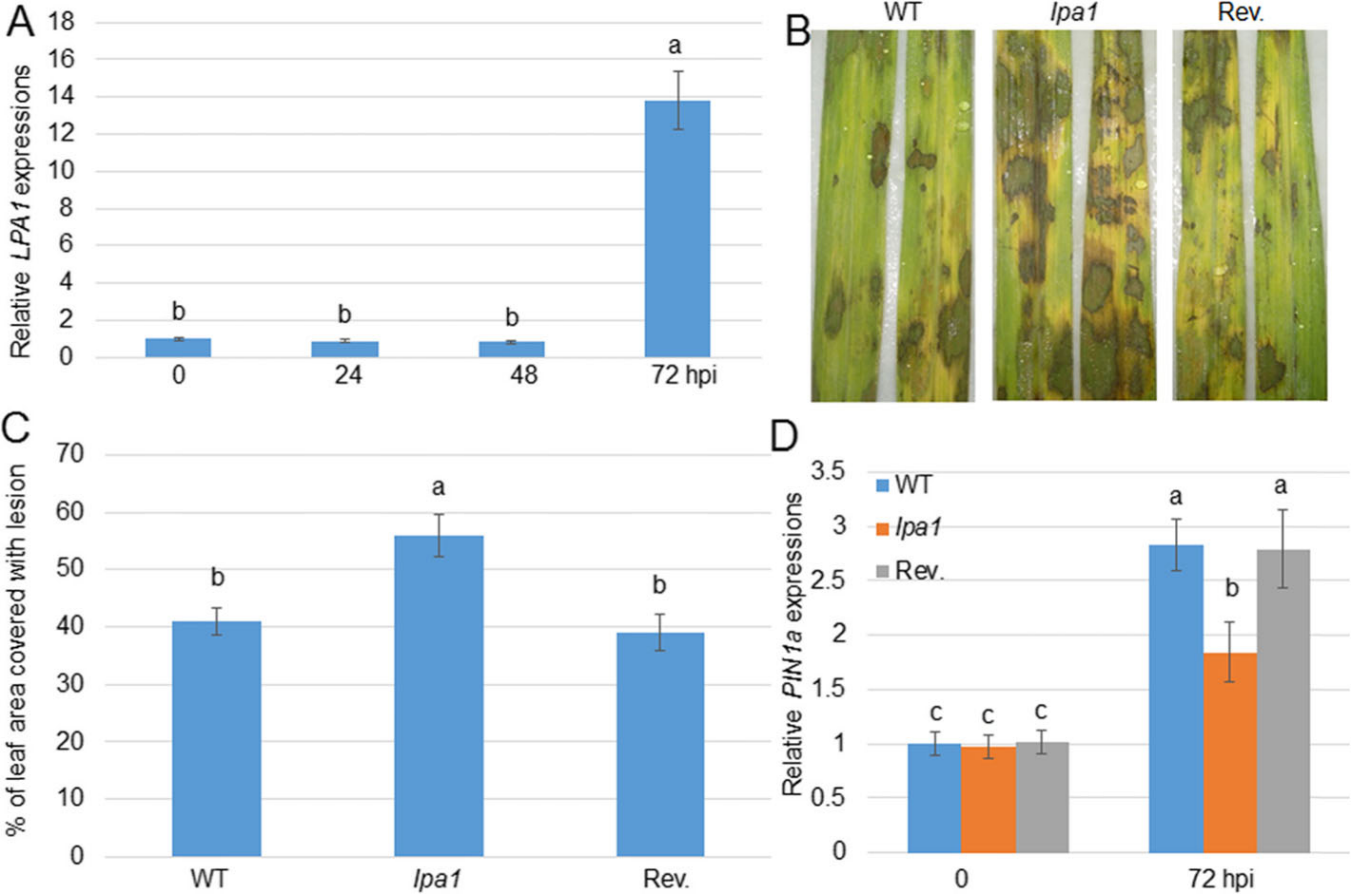
*Rhizoctonia solani*-mediated *LPA1* expression patterns and *lpa1* mutant response to sheath blight disease. **a***LPA1* expression level in the leaves at 0, 24, 48, and 72 h post-inoculation (hpi) of *R. solani* AG1-IA. The error bars are the mean ± SE (*n* = 3). **b** Response of *lpa1* and revertant (Rev.) to *R. solani* AG1-IA compared with the wild-type (WT). **c** Percentage of the leaf area covered with lesions in *lpa1* and revertant (Rev.) compared with the WT. Data represent means ± SE (*n* > 10). **d***R. solani*-mediated expression of *PIN1a* in WT, *lpa1*, and Rev. leaves before and after 72 hpi of *R. solani*. Different letters indicate significant differences at *P < 0.05*

Previously we demonstrated that the overexpression of *LPA1* significantly promotes the resistance of rice to ShB via the activation of *PIN1a* (Sun et al. 2019). However, previous research indicated that *LPA1* was expressed at very low levels in the leaves and sheath (Wu et al. 2013). Therefore, we analyzed the *R. solani* infection-dependent *LPA1* expression in more detail. Interestingly, infection with *R. solani* significantly induced *LPA1* expression after 72 h (Fig. 1a). Examination of the response of *lpa1*, *LPA1* revertant (Rev.), and wild-type (WT) plants (Liu et al. 2016) showed that *lpa1* was more susceptible to *R. solani* AG1-IA than the WT and revertant (Rev.) plants (Fig. 1b). The percentage of the leaf area covered with lesions was 41% in the WT, 56% in *lpa1*, and 39% in Rev. (Fig. 1c). Since LPA1 activates *PIN1a* transcription, the *PIN1a* expression level was examined in the wild-type, *lpa1*, and Rev. plants before and after inoculation with *R. solani* AG1-IA. The qRT-PCR results showed that *PIN1a* was induced by *R. solani* AG1-IA inoculation, and the level was lower in *lpa1* than in the wild-type and Rev. plant leaves 72 h post-*R. solani* inoculation, but there were no significant differences without the inoculation (Fig. 1d).

### IDD13 and IDD3 Interact with LPA1

**Fig. 2:**
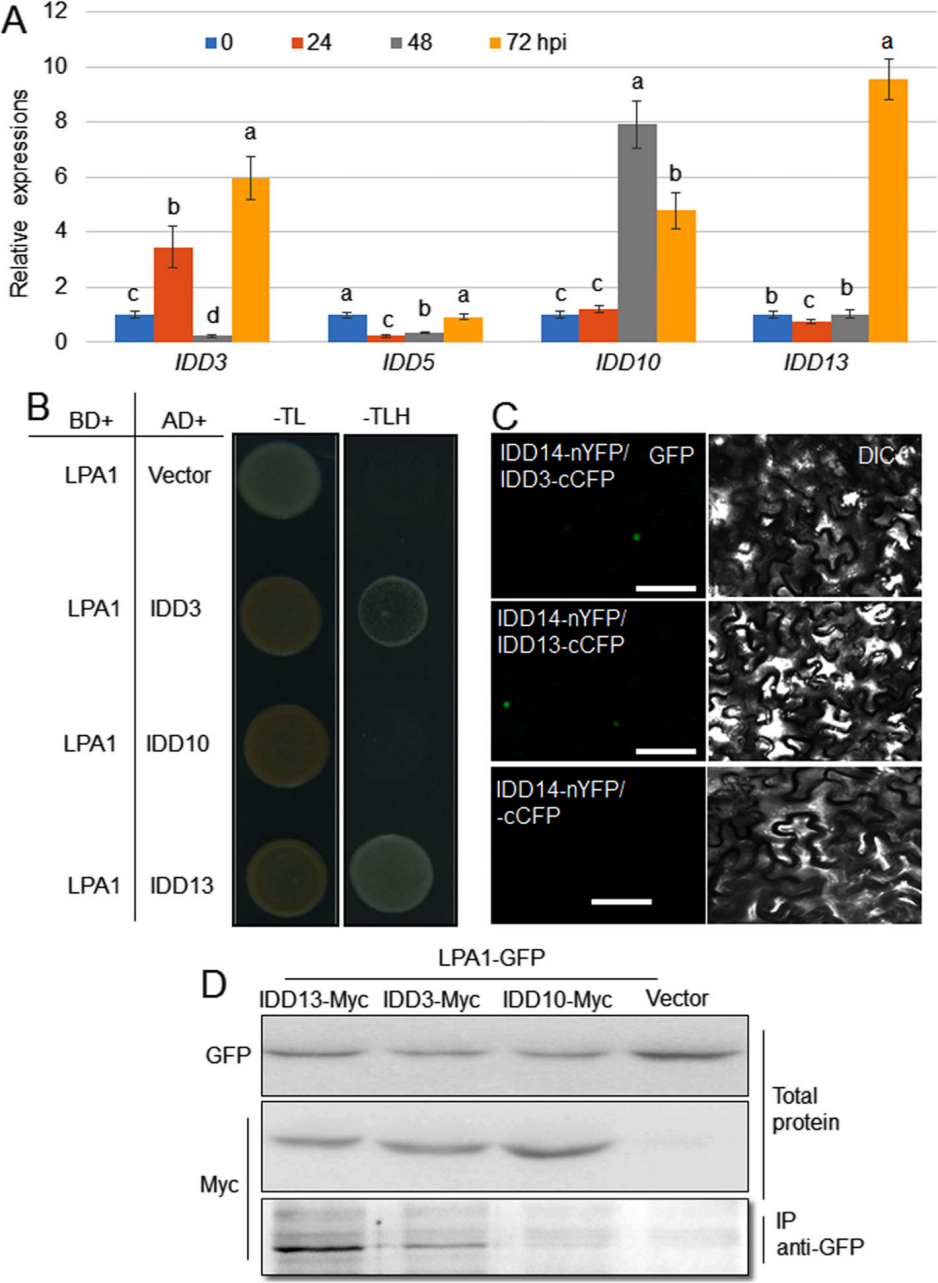
Interaction between LPA1 and IDD3 or IDD13. **a***IDD3*, *IDD5*, *IDD10*, and *IDD13* expression levels in the leaves at 0, 24, 48, and 72 h post-inoculation (hpi) of *Rhizoctonia solani* AG1-IA. The error bars are the mean ± SE (*n* = 3). The statistical analysis was performed for each gene, and different letters indicate significant differences at *P < 0.05*. **b** A yeast two-hybrid assay was performed to analyze the interaction between LPA1 and IDD3, IDD10, or IDD13. BD: GAL4-DNA binding domain; AD: activation domain; −T: without tryptophan; −L: without leucine; −H: without histidine. **c** Reconstitution of GFP fluorescence from LPA1-nYFP + IDD3-cCFP, LPA1-nYFP + IDD13-cCFP, and LPA1-nYFP + cCFP. Bars = 10 μm. DIC: differential interference contrast. **d** A co-IP assay was performed to analyze the interaction between LPA1 and IDD3, IDD13, or IDD10 in tobacco leaves. IDD3-Myc, IDD13-Myc, IDD10-Myc + LPA1-GFP, or empty vector + LPA1-GFP were transformed into tobacco leaves using Agrobacterium-mediated transformation. Green fluorescent protein (GFP) antibody-immunoprecipitated proteins were analyzed using western blot analysis with the Myc antibody. IDD3-Myc, IDD13-Myc, IDD10-Myc, and LPA1-GFP levels were analyzed by western blot using Myc and GFP antibodies, respectively

Our transcriptome study discerned that several IDD genes, including *IDD3*, *IDD5*, *IDD10*, and *IDD13*, were differentially expressed upon *R. solani* infection (unpublished data). To verify the transcriptome data, qRT-PCR was performed to examine the expression of the *IDD* gene. The results showed that *IDD5* was suppressed, while *IDD3*, *IDD10*, and *IDD13* were induced by *R. solani* (Fig. 2a). To test whether IDD3, IDD10, or IDD13 interact with LPA1, yeast two-hybrid, split-GFP, and co-immunoprecipitation (co-IP) assays were performed. A yeast-two hybrid analysis indicated that LPA1 interacts with IDD3 and IDD13 but not with IDD10 (Fig. 2b). An additional split-GFP assay showed that LPA1 interacts with IDD3 or IDD13 at the nucleus in *N. benthamiana* leaves, but the negative control (LPA1-nYFP+cCFP) did not exhibit a visible signal (Fig. 2c). For the co-IP assay, LPA1-GFP was co-expressed with IDD3-Myc, IDD13-Myc or IDD10-Myc in *N. benthamiana* leaves, and the total proteins were immunoprecipitated using an anti-GFP antibody. The immunoprecipitated proteins were analyzed using an anti-Myc antibody. The results indicated that LPA1 interacts with IDD3 and IDD13 but not IDD10 in plants, and the interaction affinity in LPA1-IDD13 was higher than that in LPA1-IDD3 (Fig. 2d).

### IDD3 Negatively and IDD13 Positively Regulated Resistance to Sheath Blight Disease

**Fig. 3:**
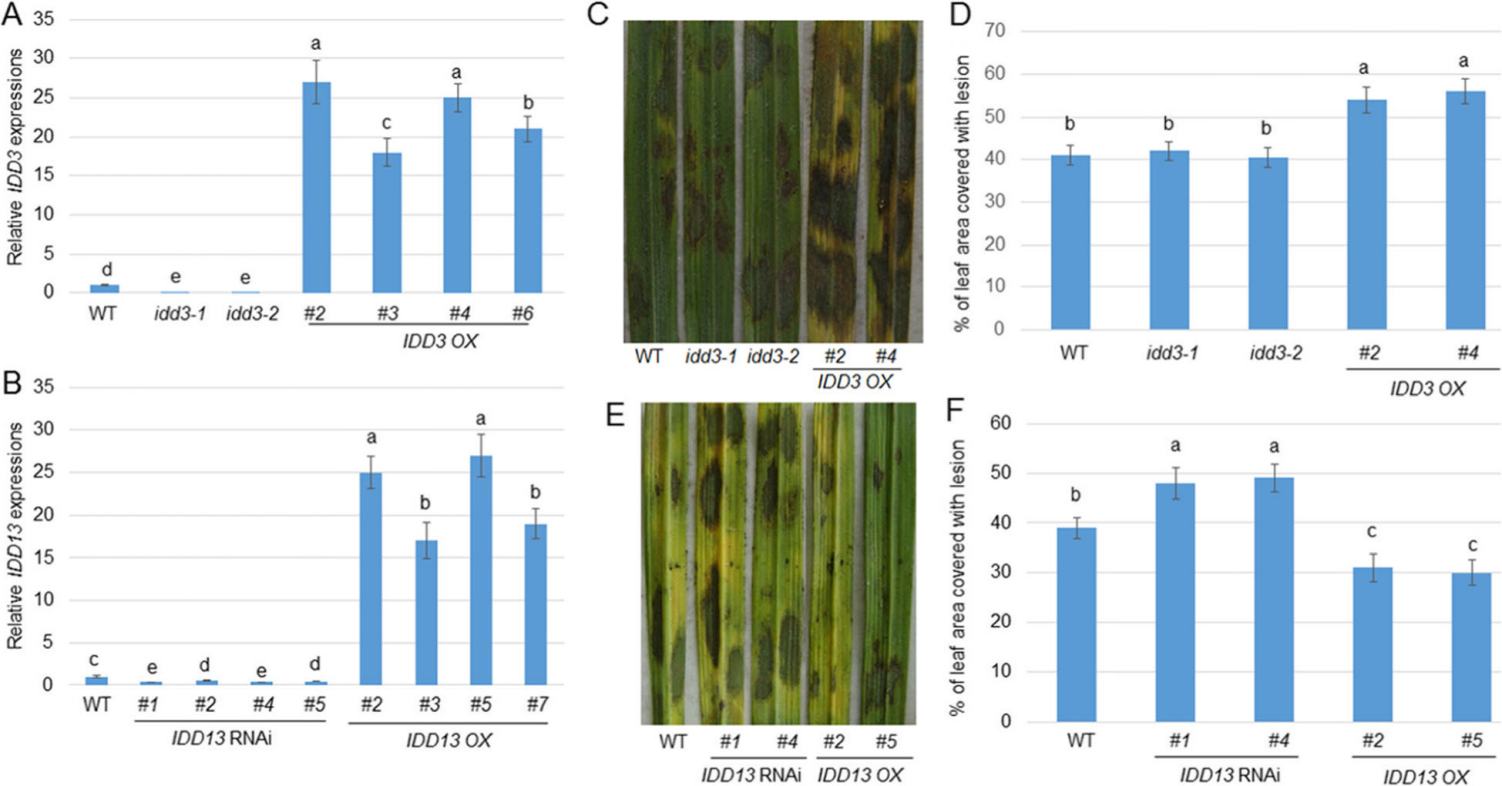
Response of *IDD3* and *IDD13* mutants to sheath blight (*Rhizoctonia solani)*. **a***IDD3* expression level was examined in the wild-type, the *idd3* mutants (*idd3–1* and *idd3–2*), and *IDD3 OX* (*#2*, *#3*, *#4*, and *#6*) plants. The error bars are the mean ± SE (*n* = 3). **b** IDD13 expression level was detected in the wild-type, *IDD13 RNAi* (*#1*, *#2*, *#4*, and *#5*), and *IDD13 OX* (*#2*, *#3*, *#5*, and *#7*) plants. The error bars are the mean ± SE (*n* = 3). **c** Response of the *idd3* mutants and *IDD3 OX* (*#2* and *#4*) plants to *R. solani* AG1-IA compared with the wild-type (WT). **d** Percentage of leaf area covered with lesions in the lines shown in (c). Data represent the means ± SE (*n* > 10). **e** Response of *IDD13 RNAi* (*#1* and *#4*) and *IDD13 OX* (*#2* and *#5*) plants to *R. solani* AG1-IA compared with the wild-type (WT). **f** Percentage of leaf area covered with lesions in the lines shown in (e). Data represent the means ± SE (n > 10). Different letters indicate significant differences at *P < 0.05*

To analyze the function of IDD3 and IDD13 in response to ShB, *idd3* mutants (*idd3–1* and *idd3–2*), *IDD3* overexpressors (OX), *IDD13 RNAi*, and *IDD13 OX* plants were tested. Before examining their response to ShB, the levels of expression of *IDD3* and *IDD13* were analyzed. The qRT-PCR results showed that the *IDD3* transcript was not detected in two *idd3* knock-out mutants (*idd3–1* and *idd3–2*), while *IDD3* was highly expressed in the *IDD3 OX* plants (*#2*, *#3*, *#4*, and *#6*) compared with the wild-type control (Fig. 3a). In addition, *IDD13* was significantly suppressed in the *IDD13 RNAi* lines (*#1*, *#2*, *#4*, and *#5*), while it was obviously highly expressed in the *IDD13 OX* plants (*#2*, *#3*, *#5*, and *#7*) compared with the wild-type control (Fig. 3b). An additional *R. solani* infection test showed that the *idd3* mutants were similar to the wild-type control and displayed a susceptible response to *R. solani* AG1-IA, but *IDD3 OX* exhibited more susceptible symptoms than those in the wild-type plants. The percentage of the leaf area covered with lesions was 41% in WT, 42% in *idd3–1*, 40.5% in *idd3–2*, 54.5% in *IDD3 OX #2*, and 56% in *IDD3 OX #4* plants (Fig. 3c and d). In addition, the *R. solani* infection results indicated that the *IDD13 RNAi* plants were more susceptible, while the *IDD13 OX* plants were less susceptible to ShB than the wild-type control. The percentage of leaf area covered with lesions was 39% in the WT, 48% in *IDD13 RNAi #1*, 49% in *IDD13 RNAi #4*, 31% in *IDD13 OX #2*, and 30% in *IDD13 OX #5* plants (Fig. 3e and f).

### IDD3 and IDD13 Directly Regulate *PIN1a* Transcription

**Fig. 4:**
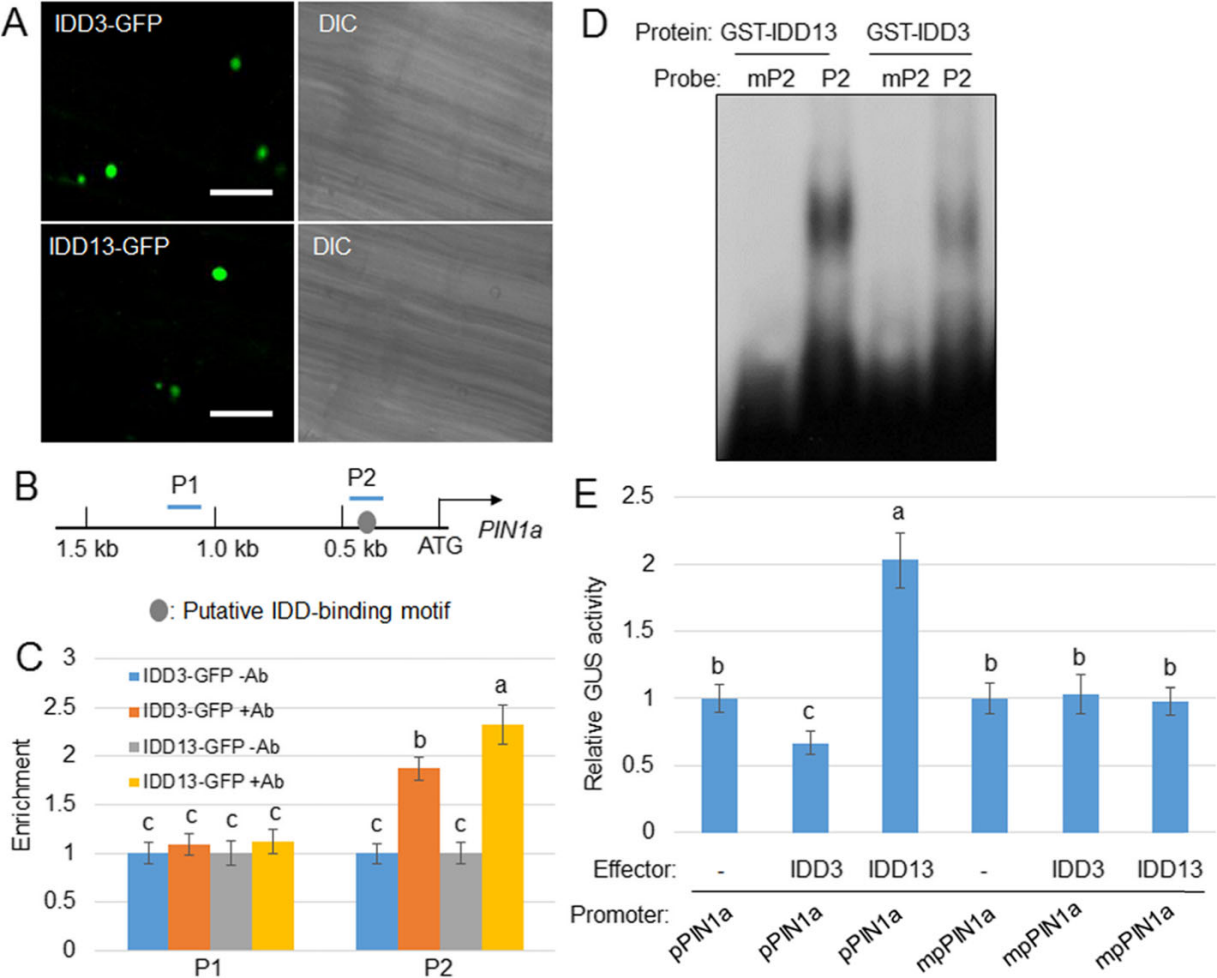
IDD3 and IDD13 bind and activate the *PIN1a* promoter. **a** IDD3-GFP and IDD13-GFP were detected in the lateral roots. GFP signal and bright field are shown in the left and right, respectively. Bars = 20 μm. **b** Schematic diagram indicating the location of the putative IDD-binding motif (gray circle) within 1.5 kb of the *PIN1a* promoter and the probes (P) used for chromatin immunoprecipitation (ChIP) assays. **c** Relative ratios of immunoprecipitated DNA to input DNA were determined by qPCR. Input DNA was used to normalize the data. –Ab or + Ab: green fluorescent protein (GFP) antibody. Error bars represent the mean ± SE (*n* = 3). **d** An electrophoretic mobility-shift assay (EMSA) was conducted to evaluate GST-IDD3 and GST-IDD13 affinities to P2 and mutated probe mP2. **e** A transient expression assay was conducted by co-transfection with *p35S:IDD3* or *p35S:IDD13* and each of the vectors expressing *GUS* under the control of native (*pPIN1a*) and IDD-binding motif-mutated (*mpPIN1a*) *PIN1a* promoters in protoplast cells. The luciferase gene driven by the 35S promoter was used as an internal control to normalize GUS expression. Error bars represent the mean ± SE (*n* = 6). Different letters indicate significant differences at *P < 0.05*

LPA1 promotes the resistance of rice to ShB via the activation of *PIN1a* (Sun et al., 2019), and IDD3 and IDD13 interact with LPA1 to regulate the resistance to ShB. Therefore, we tested the potential of IDD3 and IDD13 to bind to the *PIN1a* promoter in more detail using a chromatin immunoprecipitation (ChIP) assay. Before performing the ChIP assay, the IDD3-GFP and IDD13-GFP localization in the transgenic plants was evaluated. The GFP signal was detected in the nucleus of *IDD3-GFP* and *IDD13-GFP* transgenic lateral roots (Fig. 4a). In the *PIN1a* promoter region, a single IDD-binding motif was identified (Fig. 4b). To examine whether IDD3 and IDD13 bind to the IDD-binding motif, a ChIP assay was performed using *35S:IDD3:GFP* or *35S:IDD13:GFP* transgenic plant calli and an anti-GFP antibody. The samples without the application of the GFP antibody (−Ab) were used as the control for the GFP antibody (+Ab) to immunoprecipitate the DNA. The ChIP-PCR results showed that IDD3 and IDD13 bound to the P2 but not to the P1 (Fig. 4c). An electrophoretic mobility shift assay (EMSA) was performed to verify that IDD3 and IDD13 bound the P2 fragment. The results indicated that IDD3 and IDD13 bound to P2, but the complex failed to bind to the mutated putative IDD-binding motif (TTTGTCG mutated to AAAAAAA) mP2 (Fig. 4d). To verify the IDD3 and IDD13 activation of *PIN1a* via binding to the P2 region in the promoter, transient expression assays were conducted using the protoplast system. Protoplast cells were co-transformed with the *35S:IDD3* or *35S:IDD13* plasmid and the construct expressing the ß-glucuronidase gene (GUS) under the control of *pPIN1a* or *mpPIN1a*. In the mutated promoter (*mpPIN1a*), the IDD-binding motif sequences TTTGTCG were replaced with AAAAAAA. Protoplast cells expressing IDD13 had approximately twice the levels of activated *pPIN1a*. However, IDD13 was unable to activate *mpPIN1a*. In parallel, IDD3 suppressed *pPIN1a* by approximately one third but did not affect *mpPIN1a* (Fig. 4e).

In addition, the *PIN1a* expression level was examined in the *idd3* mutants and *IDD3 OX*, as well as in the *IDD13 RNAi* and *IDD13 OX* plants. The qRT-PCR results showed that the *PIN1a* level was obviously lower in *IDD3 OX* than in the wild-type and *idd3* mutants, but there were no significant differences between the wild-type and *idd3* mutants (Additional file 1: Figure S1a). Moreover, the *PIN1a* level was slightly lower in the *IDD13 RNAi* plants, while it was significantly higher in the *IDD13 OX* plants than in the wild-type control (Additional file 1: Figure S1b).

### IDD3 Inhibits the LPA1-Mediated Activation of *PIN1a* Expression

**Fig. 5:**
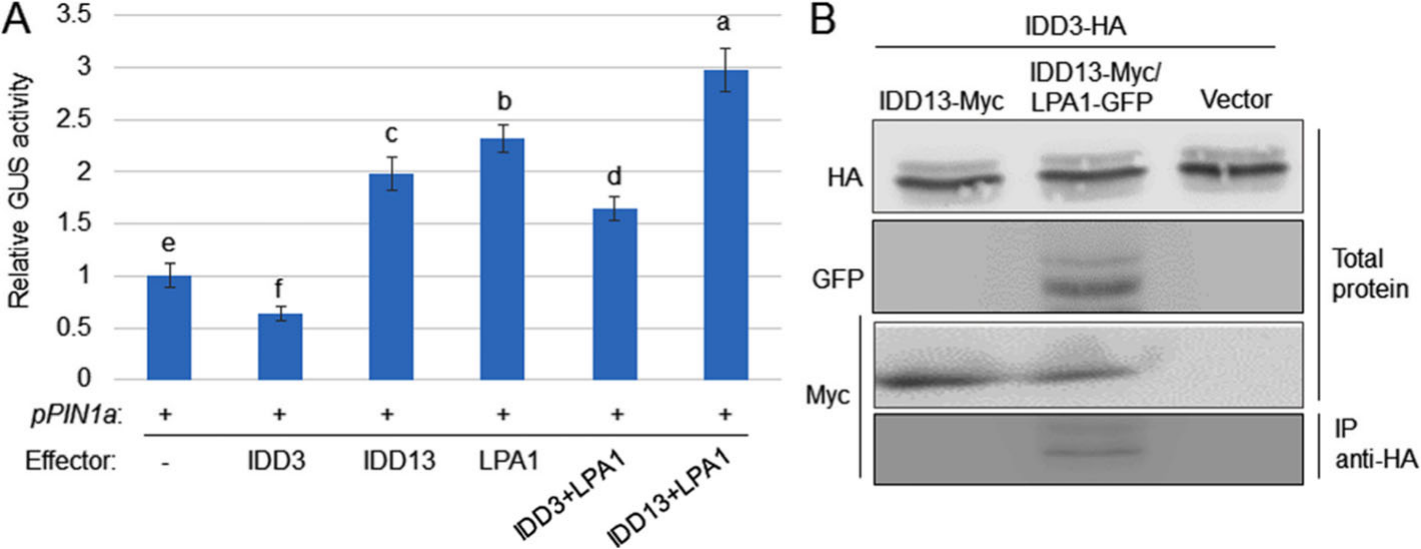
IDD3, IDD13, and LPA1 form a complex to regulate *PIN1a* transcription. **a** A transient expression assay was conducted by co-transfection with *p35S:IDD3*, *p35S:IDD13*, *p35S:LPA1*, *p35S:IDD3 + p35S:LPA1*, *p35S:IDD13 + p35S:LPA1* and the vector expressing the *GUS* under the control of native (*pPIN1a*) *PIN1a* promoters in protoplast cells. The luciferase gene driven by the 35S promoter was used as an internal control to normalize the GUS expression. Error bars represent the mean ± SE (*n* = 6). Different letters indicate significant differences at *P < 0.05*. **b** IDD3-HA + IDD13-Myc, IDD3-HA + IDD13-Myc + LPA1-GFP, or IDD3-HA + empty vector were transformed into tobacco leaves using Agrobacterium-mediated transformation. HA antibody-immunoprecipitated proteins were analyzed using western blot analysis with the Myc antibody. IDD3-HA, IDD13-Myc, and LPA1-GFP levels were analyzed by a western blot using HA, Myc, and GFP antibodies, respectively

IDD3 and IDD13 interact with LPA1, and IDD13 and LPA1 directly activate *PIN1a* transcription, while IDD3 suppresses it. Therefore, the effects of IDD3 on IDD13 and LPA1 regulation on *PIN1a* expression were examined. To verify the effect, *35S:LPA1* was co-transformed with *35S:IDD13* or *35S:IDD3* and a vector expressing *GUS* under the control of *pPIN1a*. The results indicated that IDD13 and LPA1 activated *pPIN1a*, while IDD3 suppressed *pPIN1a*. In addition, co-expressing IDD13 and LPA1 increased the activation of *pPIN1a* compared to expressing the single IDD protein. However, the expression of IDD3 inhibited the LPA1 activation of *pPIN1a* (Fig. 5a). In addition, the possibility that IDD3, IDD13, and LPA1 form a transcriptional complex was tested. IDD3-HA and IDD13-Myc were expressed in *N. benthamiana* leaves and immunoprecipitated using an anti-HA antibody, but the western blot results indicated that IDD3 did not interact with IDD13 (Fig. 5b). Additional IDD3-HA, IDD13-Myc, and LPA1-GFP proteins were expressed in *N. benthamiana* leaves, and the total protein was immunoprecipitated with an anti-HA antibody. The Co-IP results showed that IDD3, IDD13, and LPA1 form a transcriptional complex in plants (Fig. 5b).

### IDD13 Additively Functions with LPA1 in the Regulation of Resistance to Sheath Blight Disease

**Fig. 6:**
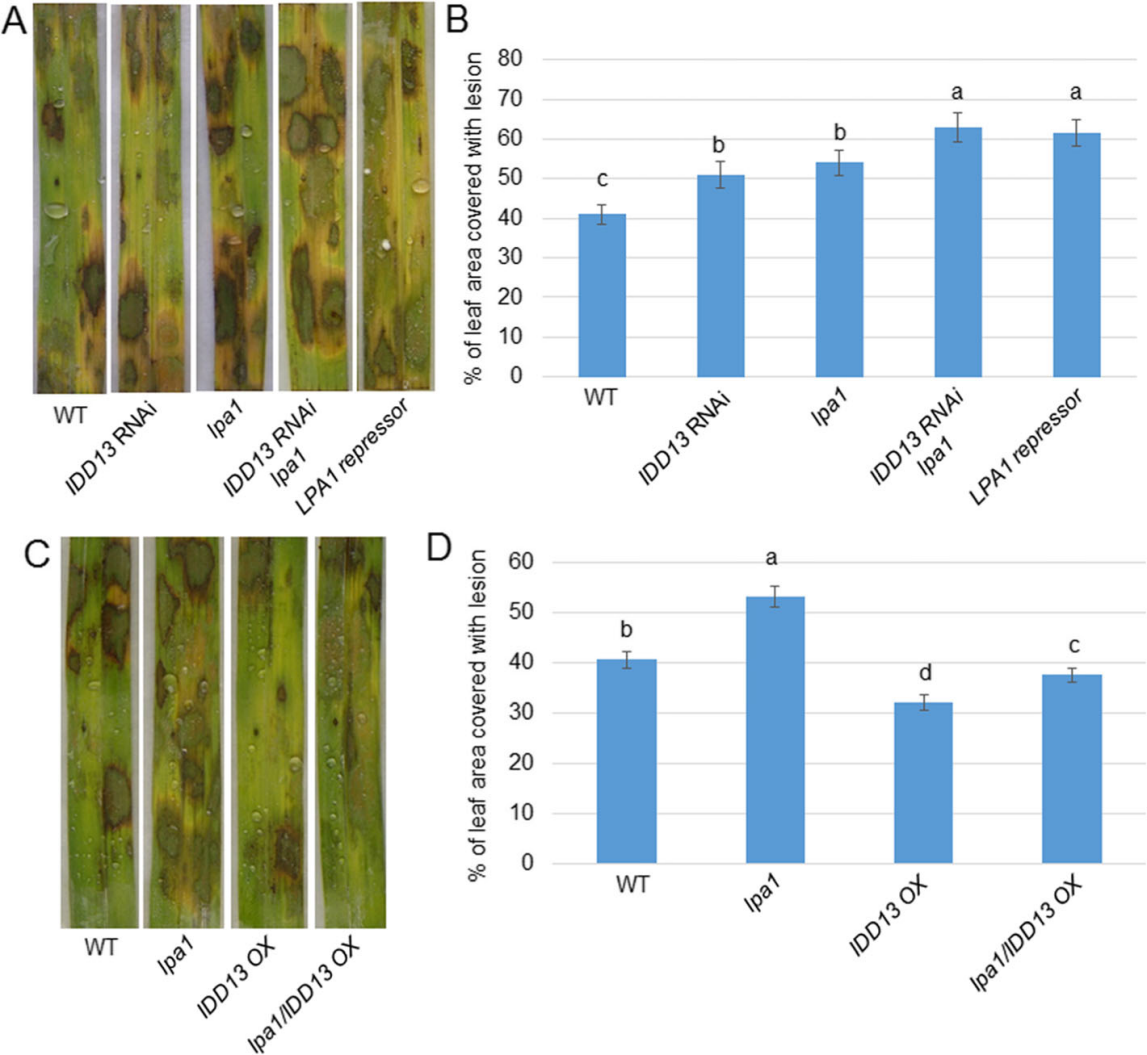
*IDD13* and *LPA1* genetic combinations in response to sheath blight (*Rhizoctonia solani*). **a** Response of *IDD13 RNAi*, lpa1, *IDD13 RNAi*/*lpa*, *LPA1* repressor plants to *R. solani* AG1-IA compared with the wild-type (WT). **b** Percentage of the leaf area covered with lesions in the lines shown in (a). Data represent the means ± SE (n > 10). **c** Response of the *lpa1*, *IDD13 OX*, and *lpa1/IDD13 OX* plants to *R. solani* AG1-IA compared with the wild-type (WT). **d** Percentage of the leaf area covered with lesions in the lines shown in (c). Data represent the means ± SE (n > 10). Different letters indicate significant differences at *P < 0.05*

The *IDD13 RNAi* and *lpa1* mutants were more susceptible to ShB, while the *idd3* mutants exhibited no significant differences compared to the wild-type control, suggesting that *IDD13* and *LPA1* but not *IDD3* might play a major role in the resistance of rice to ShB. To analyze whether *IDD13* and *LPA1* are functionally additive in the regulation of the resistance of rice to ShB, two genetic combinations were generated, including *IDD13 RNAi/lpa1* and *lpa1/IDD13 OX*. In addition, *LPA1* repressor lines were examined (Wu et al. 2013, Liu et al. 2016). An *R. solani* infection test showed that *IDD13 RNAi/lpa1* was more susceptible than the *IDD13 RNAi*, *lpa1*, and wild-type plants segregated from the same sibling, and *IDD13 RNAi/lpa1* exhibited similar susceptible symptoms to the *LPA1* repressor (Fig. 6a). The percentage of the leaf area covered with lesions was 41% in the WT, 51% in *IDD13 RNAi*, 54% in *lpa1*, 63% in *IDD13 RNAi/lpa1*, and 61.5% in the *LPA1* repressor plants (Fig. 6b). In addition, *R. solani* infection results indicated that the *lpa1/IDD13 OX* plants were less susceptible to ShB than the *lpa1* mutant and wild-type segregated from the same sibling, but they were more susceptible to ShB compared to the *IDD13 OX* plants segregated from the same sibling (Fig. 6c). The percentage of leaf area covered with lesions was 40.5% in WT, 53% in *lpa1*, 32% in *IDD13 OX*, and 37.5% in *lpa1/IDD13 OX* plants (Fig. 6d).

In addition, the level of expression of *PIN1a* was examined in the *IDD13 RNAi/lpa1*, *lpa1/IDD13 OX*, and *LPA1* repressor lines. The qRT-PCR results showed that the *PIN1a* level was much lower in *IDD13 RNAi/lpa1* than in the wild-type, *IDD13 RNAi*, and *lpa1* and was similar between *IDD13 RNAi/lpa1* and the *LPA1* repressor after *R. solani* inoculation (Additional file 2: Figure S2a). In parallel, the *PIN1a* level was higher in *lpa1/IDD13 OX* than in *lpa1* and higher than in the wild-type plants. The *PIN1a* level was noticeably higher in *IDD13 OX* than in the wild-type and *lpa1/IDD13 OX* after *R. solani* inoculation (Additional file 2: Figure S2b).

### *IDD13* Overexpresssion Maintained Yield Production in Rice

**Fig. 7:**
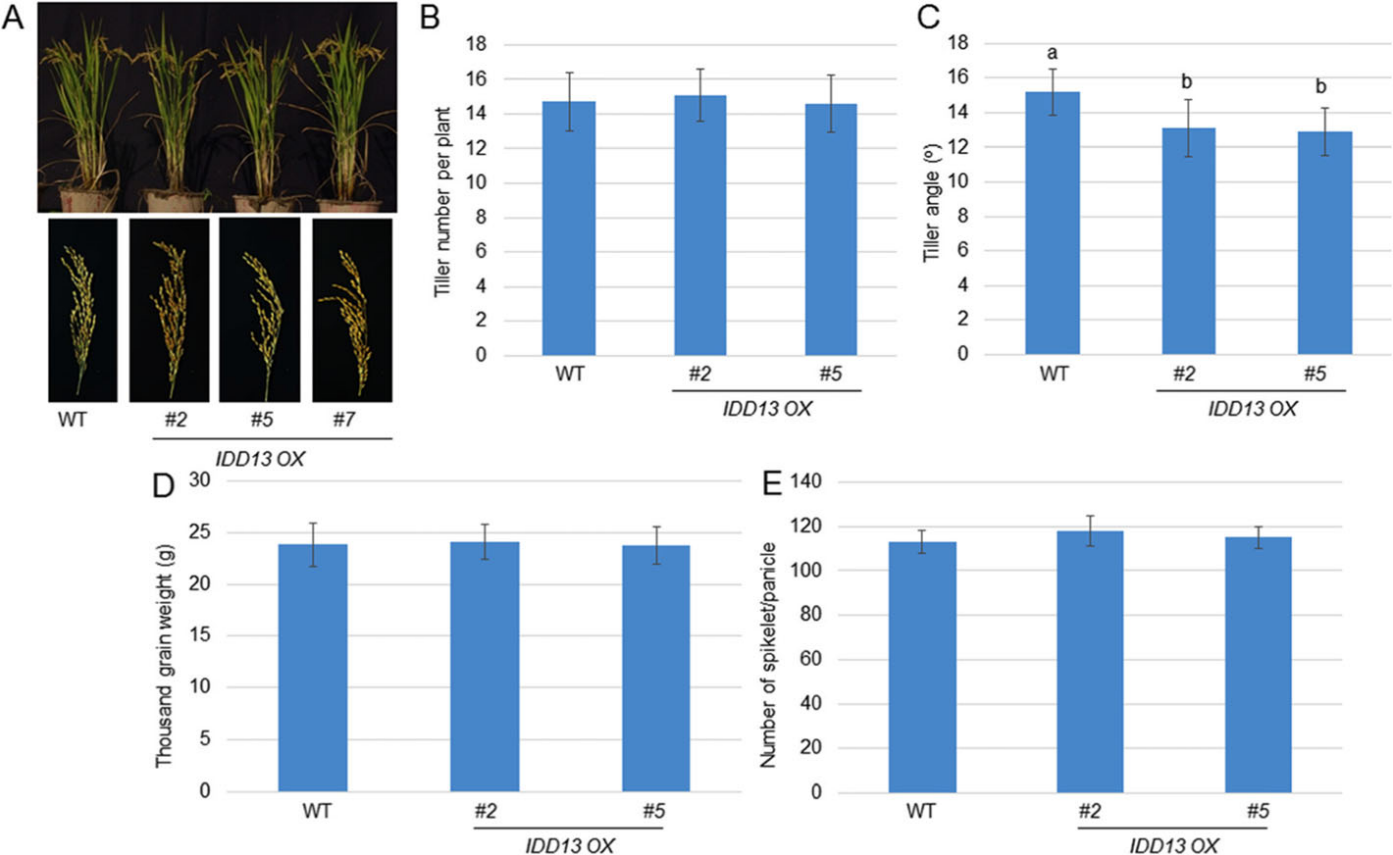
The tiller number, thousand-grain weight and number of spikelet per panicle in the wild-type and *IDD13 OX* plants. **a** The three-and-a-half-month-old wild-type and *IDD13 OX* plants (*#2*, *#5*, and *#7*), as well as their panicles, are shown. Tiller number **b**, tiller angles **c**, thousand-grain weight **d**, and the number of grain weight per panicle **e** from the wild-type and *IDD13 OX* plants (*#2* and *#5*) were calculated. Data indicate the average ± SD (*n* > 15). Different letters indicate significant differences at *P < 0.05*

Since the *IDD13 OX* plants demonstrated increased resistance to ShB, yield factors were investigated further. The results demonstrated that *IDD13 OX* plants developed a similar tiller number, thousand-grain weight, and number of spikelets per panicle relative to the WT, but the overexpression of *IDD13* slightly decreased the tiller angle compared with the wild-type (WT) plants (Fig. 7). *LPA1* overexpression increased the content of 3-indole acetic acid (IAA), a natural form of auxin, and exogenous IAA treatment promoted the resistance of rice to ShB (Sun et al. 2019). Therefore, the endogenous IAA levels in the WT, *IDD13 OX2*, and *IDD13 OX5* plants were measured. The data demonstrated that *IDD13* overexpressors contain higher levels of IAA than that of the WT plant leaves (Additional file 3: Figure S3).

## Discussion

Sheath blight disease caused by *R. solani* is a major rice disease, which severely reduces grain yield. However, the host resistance mechanisms remain unknown. Previously, we identified that exogenous auxin treatment promoted resistance to ShB, and the overexpression of *LPA1/IDD14* promoted rice defense to ShB via the activation of the auxin poplar transporter *PIN1a* in rice (Sun et al. 2019). *PIN1a RNAi* and the *PIN1a* overexpressors were more and less susceptible to ShB, respectively (Sun et al. 2019), suggesting that LPA1 increases the local auxin content or the activation of auxin signaling by controlling *PIN1a* and enhancing the resistance of the rice to ShB. However, whether IDD proteins other than LPA1 regulate the resistance of rice remains unclear.

### *IDDs* Were Induced by *R. solani*, and LPA1 Interacts with IDD3 and IDD13

The transcriptome analysis and additional qPCR verification showed that *IDD3*, *IDD10*, *IDD13*, and *LPA1* were up-regulated, while *IDD5* was down-regulated by *R. solani* in rice. In normal conditions, *LPA1* is barely expressed in the leaves and sheath of rice (Wu et al. 2013), but *R. solani* infection significantly induced the level of expression of *LPA1* in the leaves. Additional genetic study showed that the *lpa1* and *IDD13 RNAi* mutant were more susceptible, but the *idd3* mutants exhibited a similar response to ShB compared with the wild-type. In addition, the overexpression of *IDD13* produced results similar to those of *LPA1*, whereas the overexpression of *IDD3* inhibited the resistance of rice to ShB, indicating that rice defense against ShB requires *LPA1* and *IDD13*, and IDD3 negatively regulates the defense of rice to ShB. Since AtIDD15 functions in concert with AtIDD14 and AtIDD16 to directly activate auxin biosynthesis and transport-related genes in Arabidopsis (Cui et al. 2013), this suggests that IDD proteins are functionally additive in the regulation of auxin biosynthesis. Further biochemical and molecular assays identified that LPA1 interacts with IDD3 and IDD13, and the interaction affinity of LPA1 was higher with IDD13 than with IDD3*.* However, IDD3 did not directly interact with IDD13, while LPA1, IDD3, and IDD13 form a transcriptional complex, suggesting that these three IDDs may form a transcriptional complex to regulate the resistance of rice to ShB.

### IDD13 Positively and IDD3 Negatively Regulate *PIN1a*

LPA1 and IDD10 were reported to localize to the nucleus and function as transcription factors (Wu et al. 2013; Xuan et al. 2013), and IDD3-GFP and IDD13-GFP were localized to the nucleus in the transgenic rice roots. Previously, we identified that *PIN1a* is a direct target of LPA1, which positively regulates the resistance of rice to ShB (Sun et al. 2019). Since LPA1 interacts with IDD3 and IDD13, the roles of IDD3 and IDD13 in the regulation of *PIN1a* transcription were analyzed. Additional ChIP and EMAS assays showed that IDD13 and IDD3 directly bound to the putative IDD-binding motif in the *PIN1a* promoter region. In addition, a transient assay revealed that IDD13 acted similarly to LPA1 to directly activate *PIN1a*, but IDD3 directly bound to the *PIN1a* promoter and functioned as a transcriptional repressor. The transient assay results showed that the expression of IDD13 and LPA1 activated the level of transcription of *PIN1a*, while the expression of IDD3 suppressed the level of expression of *PIN1a*, suggesting that IDD13 and LPA1 function as transcriptional activators, while IDD3 functions as a transcriptional repressor to *PIN1a*.

The overexpression of *LPA1* reduced the tiller angle and increased the contents of IAA in the leaves via the activation of *PIN1a* (Sun et al. 2019). IDD13 activates *PIN1a*, and the additional investigation of yield factors identified that the overexpression of *IDD13* maintained yield production, while reducing the tiller angle compared with the wild-type plants. In addition, the *IDD13 OX* plants accumulated higher contents of IAA than were found in the wild-type plant leaves, suggesting that *IDD13 OX* acts similarly to the *LPA1 OX* plants to increase the resistance of rice to ShB by activating *PIN1a* without affecting rice production. However, the tiller angle of the *LPA1 OX* plants is smaller than that in the *IDD13 OX* plants, implying a dominant regulation of LPA1 compared with IDD13 in the transcriptional activation of *PIN1a*.

### IDD13 and LPA1 Are Functionally Additive in the Regulation of the Resistance of Rice to Sheath Blight Disease

IDD3, IDD13, and LPA1 physically interact with and differentially regulate *PIN1a*. In addition, LPA1 positively regulates the resistance of rice to ShB. Next, a genetic study was performed to analyze the functions of IDD3 and IDD13 in the control of the resistance of rice to ShB. An *R. solani* infection assay indicated that the two *idd3* mutants exhibited a similar susceptible response to ShB compared with the wild-type control. In addition, the level of expression of *PIN1a* was not changed in the *idd3* mutants, which was similar to its expression in the wild-type plants. The overexpression of *IDD3* significantly suppressed the *PIN1a* level compared with the wild-type plants, and *IDD3 OX* exhibited more susceptible symptoms to *R. solani* infection than the wild-type plants. The *IDD13 RNAi* plants were more susceptible to ShB, while the *IDD13 OX* plants were less susceptible compared with the wild-type plants. In addition, the *PIN1a* expression level was lower in the *IDD13 RNAi* and higher in the *IDD13 OX* plants than in the wild-type plants, suggesting that IDD13 might regulate the resistance to ShB via the activation of *PIN1a*. The *IDD3* mutation did not change the resistance of the rice to ShB, as well as the *PIN1a* expression, implying that IDD3 might not be a major regulator of *PIN1a* transcription.

IDD13 and LPA1 interact and activate *PIN1a* transcription. In addition, the *IDD3 RNAi* and *lpa1* mutants were more susceptible to ShB, while the *IDD13 OX* and *LPA1 OX* plants were less susceptible compared with the wild-type control. Further genetic and pathology experiments indicated that an *IDD13 RNAi/lpa1* double mutant was more susceptible to ShB compared with *IDD13 RNAi* and *lpa1*. In addition, the response of the *LPA1* repressor plants to ShB was compared with that of *IDD13 RNAi/lpa1*. The results showed that expressing the *LPA1* repressor to inhibit the transcription complex, including LPA1, produced a similar defect in response to ShB when compared with *IDD13 RNAi/lpa1*, and showed more susceptible symptom than in *lpa1* and *IDD13 RNAi*, suggesting that IDD13 and LPA1 might be functionally additive. In parallel, the *lpa1*/*IDD13 OX* double mutants were more susceptible to ShB compared with the *IDD13 OX* plants, but they were less susceptible to ShB compared to *lpa1* and the wild-type control, suggesting that *IDD13 OX* can partially rescue the defect from the *LPA1* mutation in response to ShB. Additional expression level analyses indicated that the level of *PIN 1a* was much lower in *IDD13 RNAi/lpa1* or the LPA1 repressor than in *IDD13 RNAi* and *lpa1*, while it was higher in *lpa1*/*IDD13 OX* than in *lpa1*. These results suggest that IDD13 and LPA1 might be functionally additive in the regulation of the resistance of rice to ShB via the activation of *PIN1a* expression in rice.

Overall, this study identified a new IDD transcriptional complex and identified its function in the regulation of ShB via the regulation of *PIN1a* transcription. These results will broaden our understanding of the regulatory mechanism by which the IDDs regulate auxin transport and the resistance of rice to ShB.

## Methods

### Plant Growth and *R. solani* AG1-IA Inoculation

Wild-type (WT) control line (*Oryza sativa* Japonica, cultivar Dongjin), *lpa1*, *LPA1 revertant (Rev.)*, *IDD13 RNAi*, *IDD13-GFP* overexpressor (*IDD13 OX*), *idd3–1* (PFG_3A-09378), *idd3–2* (PFG_3A-14,411), *IDD3-GFP overexpressor* (*IDD3 OX*), *lpa1/IDD13 RNAi*, *lpa1/IDD13 OX*, and *LPA1* repressor plants were used. The plants were grown in a greenhouse at Shenyang Agricultural University, China, with a temperature of 23 °C–30 °C. One-month-old rice plants were inoculated with *R. solani* AG1-IA (Prasad and Eizenga, 2008). In brief, a 10-cm-long piece was cut from the second youngest leaf of the main tiller and placed on moistened filter paper in a Petri dish (diameter, 36 cm; height, 2.5 cm). Each replicate comprised six leaves, and four replicates per line were used in a completely randomized design. Colonized potato dextrose agar (PDA) blocks (diameter, 7 mm) were excised using a circular cutter and placed on the abaxial surface of each leaf piece. The leaves were incubated at 25 °C for 72 h in a chamber with continuous light. The filter paper was kept moist with sterile water. After 72 h, the length and width of the lesions within each leaf piece were measured using Image J Fiji software (NIH, Bethesda, MD, USA) and the approximate percentage of the leaf covered with lesions was calculated as previously described (Prasad and Eizenga, 2008; Eizenga et al. 2002). To analyze the *R. solani* AG1-IA infection-mediated expression of the *IDD* genes, one-month-old wild-type plants were inoculated with *R. solani* AG1-IA, and their leaves were sampled after 0, 24, 48, and 72 h of inoculation. The accession numbers in Genbank are as follows: IDD3 (EEC85036), IDD5 (XP_015647948), IDD10 (KAB8096499), IDD13 (XP_015610838), and LPA1 (IDD14) (XP_015629419).

### RNA Extraction and Quantitative Real-Time (qRT)-PCR Analysis

Total RNA was isolated from the one-month-old rice leaves using the TRIzol reagent (Takara, Dalian, China), and the genomic DNA was removed by treatment with RQ-RNase free DNase (Promega, Madison, WI, USA). Complementary DNA was synthesized using the GoScript Reverse Transcription Kit (Promega) following the manufacturer’s instructions. A BIO-RAD CFX96 Real-time PCR system (Bio-Rad, Hercules, CA, USA) and SYBR-Green (Takara) were used for the qRT-PCR analyses. The gene expression levels were normalized to that of the level of *Ubiquitin*. The primers used for qRT-PCR are listed in Additional file 4: Table S1.

### Plasmid Construction

To generate *IDD3-GFP* and *IDD13-GFP* overexpression transgenic plants, *IDD3* and *IDD13* ORF sequences were amplified and cloned into *Bgl*II and *Spe*I restriction enzyme sites of the pCAMBIA1302 binary vector, in which *IDD3* or *IDD13* coding sequences were N-terminally fused to the *GFP* coding sequences. To generate *IDD13 RNAi* plants, 300 bp of the *IDD13* coding region was cloned into *Swa*I and *Asc*I sites in the sense and *Xba*I and *BamH*I sites in the antisense orientation, respectively, in the pFGC5941 binary vector (ChromDB).

### Yeast Two-Hybrid Assay

To test the interaction between LPA1 and IDD13, IDD3 or IDD10, the Gal4 DNA-binding domain (BD) was N-terminally fused to *LPA1*, while *IDD13*, *IDD3*, or *IDD10* ORFs were cloned into the pGAD424 vector. The pair of *IDDs* was further transformed in the yeast strain PJ69-4A (Clontech, http://www.clontech.com/). Yeast cells carrying a pair of IDDs were grown on SD/Trp−/Leu- and SD/Trp−/Leu-His- plates. The sequences of the primers for cloning the *IDD13* ORF are listed in Additional file 3: Table S1.

### Split GFP Assay

The N-proximal half of YFP (nYFP) and the C-proximal half of CFP (cCFP) sequences were fused to the C-terminal sequences of LPA1 (IDD14) and C-terminal sequences of IDD3 or IDD13 in the pXNGW and pXCGW vectors, respectively. Agrobacterium cells (GV3101) harboring half of the YFP parts were mixed and then infiltrated into *Nicotiana benthamiana* leaves. Before observing the YFP signal using a confocal microscope (Olympus X1000, Japan), the tobacco plants were grown in a growth chamber for 36 to 48 h (Kim et al. 2009a).

### Co-Immunoprecipitation (co-IP) and Western Blot Analyses

IDD3-Myc + LPA1-GFP, IDD13-Myc + LPA1-GFP, IDD10-Myc + LPA1-GFP, IDD3-HA + IDD13-Myc, or IDD3-HA + IDD13-Myc + LPA1-GFP were coexpressed in *N. benthamiana* leaves, respectively. After 36 h of expression, the protein was extracted, and Co-IP assays were performed as described previously (Kim et al. 2009b). Twenty micrograms of protein from each sample were separated on a 10% SDS-PAGE gel and electrotransferred onto Immobilon-P Transfer Membranes (MILLIPORE JAPAN, Tokyo, Japan). For the subsequent western blot analysis, the following primary antibodies were used: an anti-HA antibody (1:2000; Abcam, Cambridge, MA, USA), anti-GFP antibody (1:2000; Abcam), and anti-Myc antibody (1:2000; Abcam). The membranes were incubated for an additional hour with an anti-mouse or anti-rabbit horseradish peroxidase (HRP)-conjugated secondary antibody (1:2000; Cell Signaling Technology, Danvers, MA, USA) before the signal was detected using an ECL Western Blotting Detection System (GE Healthcare, Piscataway, NJ, USA).

### Chromatin-Immunoprecipitation (ChIP) Assay

Eight grams of rice calli were collected from transgenic plants expressing *35S:IDD13-GFP* and *35S:IDD3-GFP* for the ChIP assay. The ChIP assay and subsequent ChIP-PCR assays were followed by a protocol described previously (Je et al. 2010). The primers used for the ChIP-PCR are listed in Additional file 3: Table S1.

### Electrophoretic Mobility Shift Assay (EMSA)

To produce IDD13 and IDD3 recombinant proteins, the open reading frame sequences of *IDD13* and *IDD3* were sub-cloned into the *pGEX 5X-1* expression vector, and the resulting *pGEX 5X-1-IDD13* and *pGEX 5X-1-IDD3* plasmids were used to transform *Escherichia coli* strain BL21 DE3. Recombinant proteins were harvested after a 4 h treatment with 0.5 mM isopropyl β-D-1-thiogalactopyranoside (IPTG) at 28 °C. The EMSA was performed as previously described (Je et al. 2010). The primers used to obtain the EMSA probes are listed in Additional file 3: Table S1.

### Transient Expression Assay

For the transient assay, the effector plasmids (*35S:LPA1*, *35S:IDD13*, and *35S:IDD3*) and reporter (*pPIN1a* or mutated promoter, *mpPIN1a*), as well as an internal control plasmid (*35S:LUC*), were co-transformed into protoplast cells (Yamaguchi et al. 2010). The GUS activity analyses were performed as previously described (Xuan et al. 2013). The luciferase assay was performed using a Luciferase Assay Kit (Promega), and PEG-mediated transformation and luciferase activity assays were performed as previously described (Yoo et al. 2007). The primers used for the transient assay are listed in Additional file 3: Table S1.

### IAA Measurement

The leaves from 1-month-old *IDD13 OX2*, *IDD13 OX5*, and wild-type plants were used for IAA extraction. IAA extraction and calculation methods were followed as described by Pan et al. (2010). IAA-[α, α-D2] was used as an internal standard of IAA in the experiments.

### Statistical Analyses

Statistical analyses were performed using Prism 5.0 (GraphPad, San Diego, CA, USA). For multiple lines comparison, a one-way analysis of variance (ANOVA) was performed, followed by Bonferroni’s multiple comparison tests. Differences among the samples were considered significant at *P* < 0.05.

## Supplementary information

**Additional file 1: Figure S1.***PIN1a* expression in *IDD3* and *IDD13* mutants and overexpressors. (A) Relative expression of *PIN1a* in wild-type (WT), *idd3–1*, *idd3–2*, *IDD3 OX #2*, and *IDD3 OX #4* plant leaves. (B) Relative expression of *PIN1a* in wild-type (WT), *IDD13 RNAi (#1* and *#4)*, *IDD3 OX #2*, and *IDD3 OX #5* plant leaves. The mRNA levels of the samples were normalized to that of *Ubiquitin* mRNA. Data represent the means ± standard error (*n* = 3). The expression of *PIN1a* in the WT was defined as “1”. Different letters indicate significant differences at *P < 0.05*.

**Additional file 2: Figure S2.***PIN1a* expression in *LPA1* and *IDD13* genetic combinations. (A) Relative expression of *PIN1a* in the wild-type (WT), *IDD13 RNAi*, *lpa1*, *IDD13 RNAi/lpa1*and *LPA1 repressor* plant leaves after 72 h of *Rhizoctonia solani* inoculation. (B) Relative expression of *PIN1a* in the wild-type (WT), *lpa1*, *IDD3 OX*, and *lpa1/IDD3 OX* plant leaves after 72 h of *R. solani* inoculation. The mRNA levels of the samples were normalized to that of Ubiquitin mRNA. Data represent the means ± standard error (*n* = 3). The expression of *PIN1a* in WT was defined as “1”. Different letters indicate significant differences at *P < 0.05*.

**Additional file 3: Figure S3.** Measurement of the IAA content in WT and *IDD13* overexpressors. The contents of IAA from the leaves of 1-month-old WT and *IDD13 OX* lines (*OX2* and *OX5*) were measured. Vertical bars indicate average values ± SE (*n* = 3). Different letters indicate significant differences at *P < 0.05*.

**Additional file 4: Table S1.** Primer sequences

## Data Availability

The datasets supporting the conclusions of this article are provided within the article and its additional files.

## Abbreviations

ChIP: Chromatin-Immunoprecipitation
DIC: Differential Interference Contrast
EMSA: Electrophoretic Mobility Shift Assay
IDD: Indeterminate Domain
LPA1: Loose Plant Architecture1
OX: Overexpressor
PIN1a: Pin-Formed 1a
ShB: Sheath Blight Disease
WT: Wild Type
