# Supplementary material for: Indeterminate Domain Proteins Regulate Rice Defense to Sheath Blight Disease
Source: Rice (N Y). 2020 Mar 6;13:15. doi: 10.1186/s12284-020-0371-1 (PMC7058748; doi:10.1186/s12284-020-0371-1)
Supplement: Supplementary file 1 — Additional file 1: Figure S1.PIN1a expression in IDD3 and IDD13 mutants and overexpressors. (A) Relative expression of PIN1a in wild-type (WT), idd3–1, idd3–2, IDD3 OX #2, and IDD3 OX #4 plant leaves. (B) Relative expression of PIN1a in wild-type (WT), IDD13 RNAi (#1 and #4), IDD3 OX #2, and IDD3 OX #5 plant leaves. The mRNA levels of the samples were normalized to that of Ubiquitin mRNA. Data represent the means ± standard error (n = 3). The expression of PIN1a in the WT was defined as “1”. Different letters indicate significant differences at P < 0.05. [file 12284_2020_371_MOESM1_ESM.docx]

**Supplementary Figures**

**
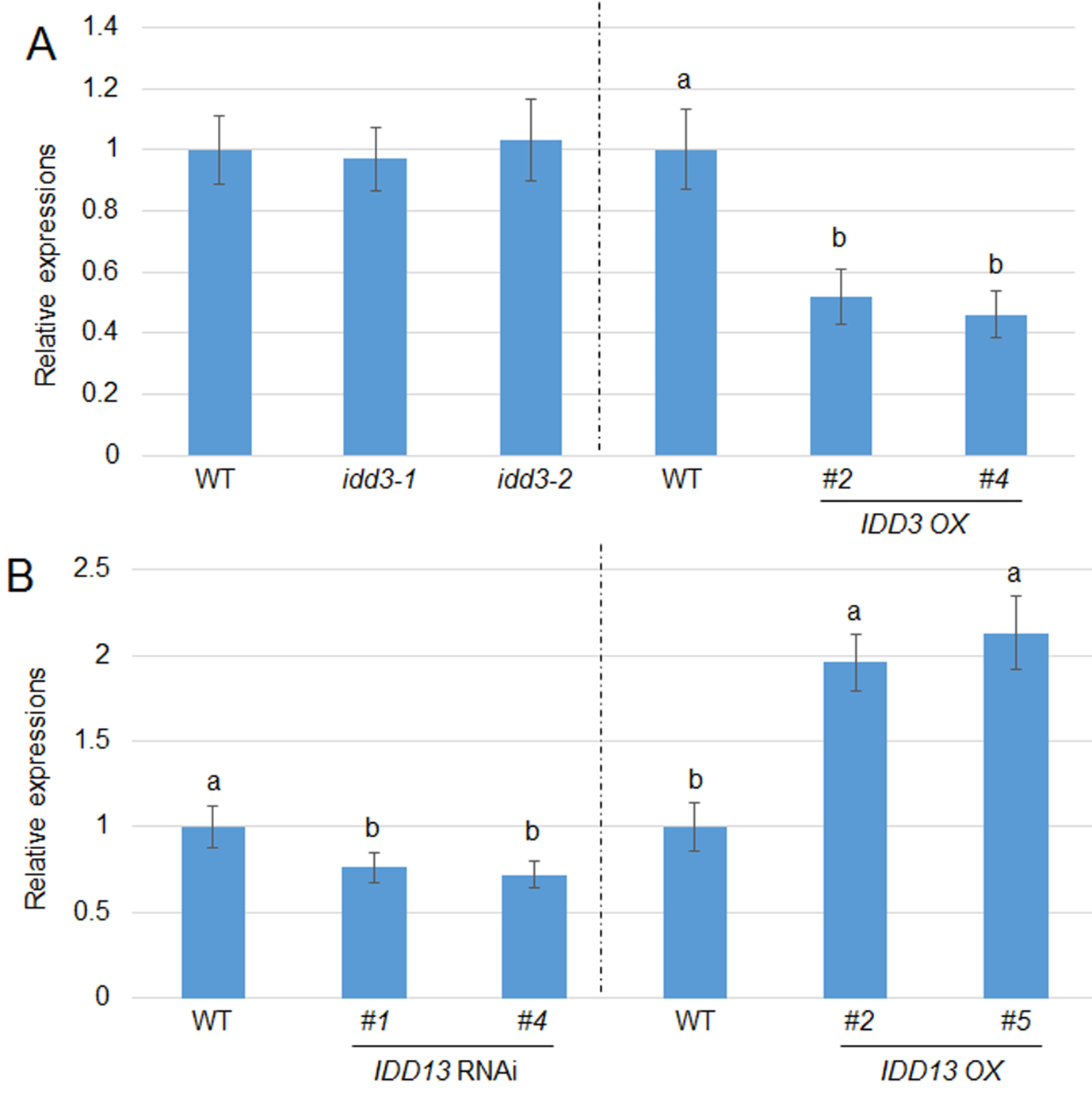
**

**Fig. S1** *PIN1a* expression in *IDD3* and *IDD13* mutants and overexpressors. (A) Relative expression of *PIN1a* in wild-type (WT), *idd3-1*, *idd3-2*, *IDD3 OX #2*, and *IDD3 OX #4* plant leaves. (B) Relative expression of *PIN1a* in wild-type (WT), *IDD13* RNAi *(#1* and *#4)*, *IDD3 OX #2*, and *IDD3 OX #5* plant leaves. The mRNA levels of the samples were normalized to that of *Ubiquitin* mRNA. Data represent the means ± standard error (n = 3). The expression of *PIN1a* in the WT was defined as “1”. Different letters indicate significant differences at *P <0.05*.
