# Supplementary material for: Indeterminate Domain Proteins Regulate Rice Defense to Sheath Blight Disease
Source: Rice (N Y). 2020 Mar 6;13:15. doi: 10.1186/s12284-020-0371-1 (PMC7058748; doi:10.1186/s12284-020-0371-1)
Supplement: Supplementary file 2 — Additional file 2: Figure S2.PIN1a expression in LPA1 and IDD13 genetic combinations. (A) Relative expression of PIN1a in the wild-type (WT), IDD13 RNAi, lpa1, IDD13 RNAi/lpa1and LPA1 repressor plant leaves after 72 h of Rhizoctonia solani inoculation. (B) Relative expression of PIN1a in the wild-type (WT), lpa1, IDD3 OX, and lpa1/IDD3 OX plant leaves after 72 h of R. solani inoculation. The mRNA levels of the samples were normalized to that of Ubiquitin mRNA. Data represent the means ± standard error (n = 3). The expression of PIN1a in WT was defined as “1”. Different letters indicate significant differences at P < 0.05. [file 12284_2020_371_MOESM2_ESM.docx]

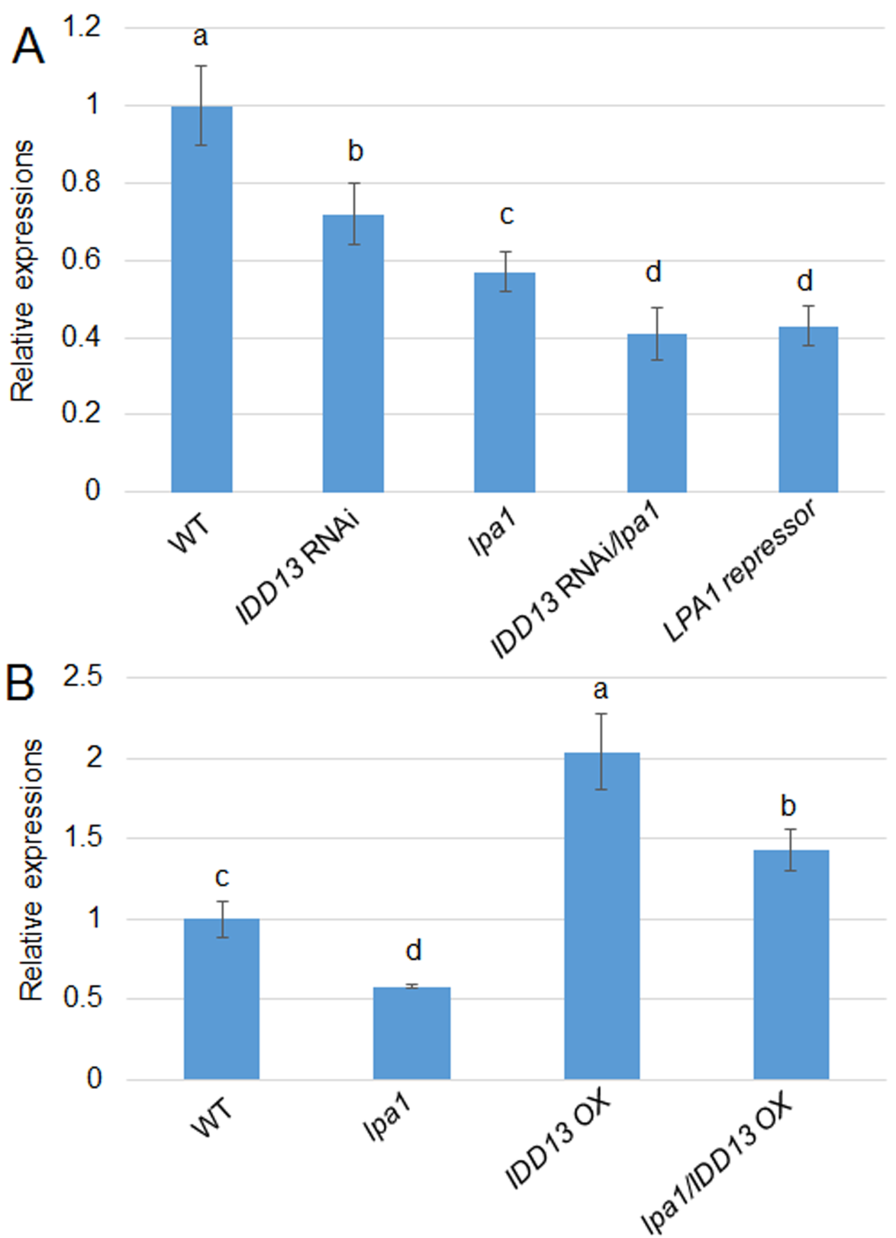


**Fig. S2** *PIN1a* expression in *LPA1* and *IDD13* genetic combinations. (A) Relative expression of *PIN1a* in the wild-type (WT), *IDD13* RNAi, *lpa1*, *IDD13* RNAi*/lpa1*and *LPA1 repressor* plant leaves after 72 hours of *Rhizoctonia solani* inoculation. (B) Relative expression of *PIN1a* in the wild-type (WT), *lpa1*, *IDD3 OX*, and *lpa1/IDD3 OX* plant leaves after 72 hours of *R. solani* inoculation. The mRNA levels of the samples were normalized to that of Ubiquitin mRNA. Data represent the means ± standard error (n = 3). The expression of *PIN1a* in WT was defined as “1”. Different letters indicate significant differences at *P <0.05*.
