# Supplementary material for: Indeterminate Domain Proteins Regulate Rice Defense to Sheath Blight Disease
Source: Rice (N Y). 2020 Mar 6;13:15. doi: 10.1186/s12284-020-0371-1 (PMC7058748; doi:10.1186/s12284-020-0371-1)
Supplement: Supplementary file 3 — Additional file 3: Figure S3. Measurement of the IAA content in WT and IDD13 overexpressors. The contents of IAA from the leaves of 1-month-old WT and IDD13 OX lines (OX2 and OX5) were measured. Vertical bars indicate average values ± SE (n = 3). Different letters indicate significant differences at P < 0.05. [file 12284_2020_371_MOESM3_ESM.docx]

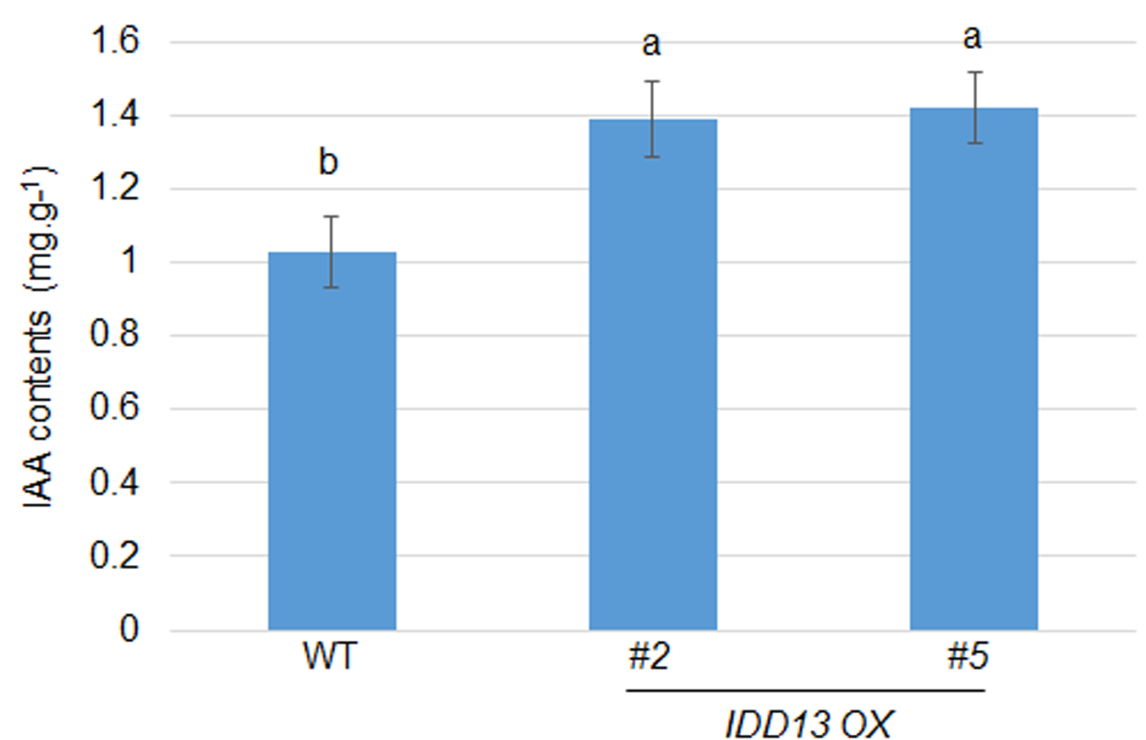


**Fig. S3.** Measurement of the IAA content in WT and IDD13 overexpressors. The contents of IAA from the leaves of 1-month-old WT and IDD13 OX lines (OX2 and OX5) were measured. Vertical bars indicate average values ± SE (n = 3). Different letters indicate significant differences at *P <0.05*.
