# Supplementary material for: Indeterminate Domain Proteins Regulate Rice Defense to Sheath Blight Disease
Source: Rice (N Y). 2020 Mar 6;13:15. doi: 10.1186/s12284-020-0371-1 (PMC7058748; doi:10.1186/s12284-020-0371-1)
Supplement: Supplementary file 4 — Additional file 4: Table S1. Primer sequences [file 12284_2020_371_MOESM4_ESM.docx]

**Supplementary Tables**

**Table S1.** Primer sequences

| Primers | Sequences |
| --- | --- |
| IDD3 RT-F | CCTCACCTGAAGCCAACAAAG |
| IDD3 RT-R | ATCACTACAGCACAGCACAG |
| IDD5 RT-F | CTGGACATGGGGTCTGCAC |
| IDD5 RT-R | TACCAATACAGGCAGGTGGC |
| IDD10 RT-F | ACCTCACCGGCATCAAGAAG |
| IDD10 RT-R | AGAGGCTGAGCGCCATGTTC |
| IDD13 RT-F | GGGCATCATGTAGCCAAAGAA |
| IDD13 RT-R | CGTCGTCTTAATTTGCTGGTG |
| LPA1 RT-F | GCGTATGTATGTAAAGCAAG |
| LPA1 RT-R | GAAACGACCTACGAAACTAC |
| PIN1 RT-F | TCTCGCTCGGACATCTACTCCC |
| PIN1 RT-R | AGTCCTCCCTGTCCTTCGCTC |
| UBQ RT-F | CAAGATGATCTGCCGCAAATGC |
| UBQ RT-R | TTTAACCAGTCCATGAACCCG |
| IDD3 GST-F | GAATTCAAGAAGAAGAGGAACCAGCC |
| IDD3 GST-R | GTCGACGGGCGGCATGCGCGCGTTC |
| IDD13 GST-F | GAATTCCCGGACGAGGCGACCACGC |
| IDD13 GST-R | GTCGACCGGCGCCATGTCGAACCGG |
| IDD13 Ri-F | TCTAGAGGCGCGGCGGCGGCGGGGGCGCCACTACC |
| IDD13 Ri-R | GGATCCATTTAAATCGATCTCGACGTCTTAATTTG |
| IDD3 Hind-F | AAGCTTATGGCGGCCGCCTCGTCCGCACCCTTC |
| IDD3 BamH-R | GGATCCCATGTTTGCCGGGTCCAGTGAGCCGAC |
| IDD13 Hind-F | AAGCTTATGTTGGGTTCTTGCGCCCCGAC |
| IDD13 BamH-R | GGATCCCATGATGCCCATGCTGTTAGCGTGCTG |
| IDD3 AD-F | GAATTCATGGCGGCCGCCTCGTCCGCACCCTT |
| IDD3 AD-R | GTCGACTCAGTTCATGTTTGCCGGGTCCAG |
| IDD13 AD-F | GAATTCATGTTGGGTTCTTGCGCCCCGAC |
| IDD13 AD-R | GTCGACCATGATGCCCATGCTGTTAG |
| IDD10 AD-F | GAATTCATGTCATCGAATTCATCGGC |
| IDD10 AD-R | GTCGACTCAGCCCTGGCTGGCATGCTC |
| LPA1 BD-F | GAATTCATGGCACTGGTCAAGAGCCACCAC |
| LPA1 BD-R | GTCGACGATGCCGGCGCCGACGCCGCCGCCCCTC |
| P1-F | AGGAATGCATGACACAGTC |
| P1-R | CACGCGCGCGTGGATGGTAC |
| P2-F | ATGGCTGATGGCTCAACG |
| P2-R | TGCCAGCTCCCTGTCCTTG |
| PIN1 P-F | AAGCTTCCGTTTCAGGAATATTCACATCTACG |
| PIN1 P-R | GGATCC CTTCGCCCCCCCTCTTCCCTCCTC |
